# Elastic properties of single-walled carbon nanotube thin film by nanoindentation test

**DOI:** 10.1038/s41598-017-11722-y

**Published:** 2017-09-12

**Authors:** Xingling Tang, Abdelkhalak El-Hami, Khalil El-Hami, Mohamed EID, Chaorun Si

**Affiliations:** 1 School of Mechatronical Engineering, Beijing Institute of Technology, Beijing, 100081 China; 2 China Nuclear Power Engineering Co., Ltd, Beijing, 100840 China; 3 LOFIMS, INSA de Rouen, Avenue de l’Universite, Saint Etienne de Rouvray, 76800 France; 4 Laboratory of Nano-sciences and Modeling, Faculty of Khouribga, Univ. Hassan 1, BP.145, Khouribga, Morocco; 5 CEA Saclay, Bat. 470, 91191Gif-sur-Yvette, Cedexe, France

## Abstract

This paper carries out a preliminary study for the elastic properties of single walled carbon nanotube (SWCNT) thin film. The SWCNT thin films (~250 nm) are prepared by a simple and cost effective method of spin-coating technology. Nanoindentation test with a Berkovich indenter is used to determine the hardness and elastic modulus of the SWCNT thin film. It is important to note that the elastic properties of SWCNT film are indirectly derived from the information of load and displacement of the indenter under certain assumptions, deviation of the ‘test value’ is inevitable. In this regard, uncertainty analysis is an effective process in guarantying the validity of the material properties. This paper carries out uncertainty estimation for the tested elastic properties of SWCNT film by nanoindentation. Experimental results and uncertainty analysis indicates that nanoindentation test could be an effective and reliable method in determine the elastic properties of SWCNT thin film. Moreover, the obtained values of hardness and elastic modulus can further benefit the design of SWCNT thin film based components.

## Introduction

Exploring the potential of carbon nanotube (CNT) films has being one of the intense researches since the fabrication and purification of thin films of CNTs became a relatively mature technique. The discovery of exceptional transparency, conductivity, and flexibility properties suggest CNT films potential applications in different fields of electronic, optoelectronic, and sensor systems^1, 2^.

Until recently, a lot of work has been done in the fabrication of thin CNT films with optimized collective electrical, optical, and mechanical properties by controlling the tube density, the overall spatial layouts, the lengths, and their orientations^3^. Wet methods such as drop drying or electrophoretic deposition have been proposed as a convenient method in preparing thin films of CNTs with controlled morphology and desired function^4, 5^. Compared with the efforts which focused on the techniques for producing well defined CNT films, little attention has been paid to the mechanical properties of these thin films even though the elastic properties could be an important issue in their ultimate application. It is demonstrated that the mechanical deformation of CNTs may cause considerable changes in its electronic, optical, magnetic, and chemical properties^6, 7^. Besides, the mechanical properties are very different for the CNT films with different chemical component or stress and strain states. Lee found that the poisson’s ratio of SWCNTs and MWNTs sheets can change from 0.06 to −0.20 as the *wt*% of MWNTs changes from 0 to 100%^8^. Yin and his colleagues discovered that the poisson’s ratio of CNT films can change from negative to positive during a uniaxial tensile loading^9^. These interest findings indicate that the mechanical properties of CNT films could be essential in fulfilling their ultimate application.

With high-resolution of load and displacement data, nanoindentation test was realized to be a very useful technique in extracting the mechanical properties of materials^10, 11^. In nanoindentation process, an indenter tip is pressed into the specimen. Penetration and force on the indenter are continuously recorded by high-resolution depth-sensing instruments. The indentation system can reaches a load and displacement resolution of several nN and less than 1 nm, respectively. In addition, nanoindentation does not require the removal of the specimen from its substrate, which greatly reduces the difficulty of specimen preparation. With those properties mentioned above, nanoindentation has been used in determining the mechanical properties of materials in nano/micro-scale and other extracted property in physical sciences^12^. Qi^13^ used nanoindentation technique and their proposed micro-mechanical model to measure the bending stiffness, wall modulus and axial modulus of the constituent nanotubes. Liu^14^ presented an atomistic study on the nanoindentation mechanisms of single-walled and multi-walled CNTs and CNT clusters. Their study demonstrated that the deformation characteristics of CNTs are related to its elastic stiffness.

Those experimental and theoretical studies suggest that nanoindentation technique could be an effective method in determine the mechanical properties of CNT films. As the importance of mechanical properties for SWCNT films in their real applications, the objective of this paper is to investigate the elastic modulus of SWCNT thin film. SWCNT thin films are prepared by using the easy operating and time-saving method of spin-coating technique. The hardness and elastic modulus of SWCNT film are estimated by nanoindentation test. Uncertainty analysis for the tested results indicates that nanoindentation test is a reliable and effective method in determining the elastic properties of SWCNT thin film.

## Experimental Details

### Material preparation

**Figure 1:**
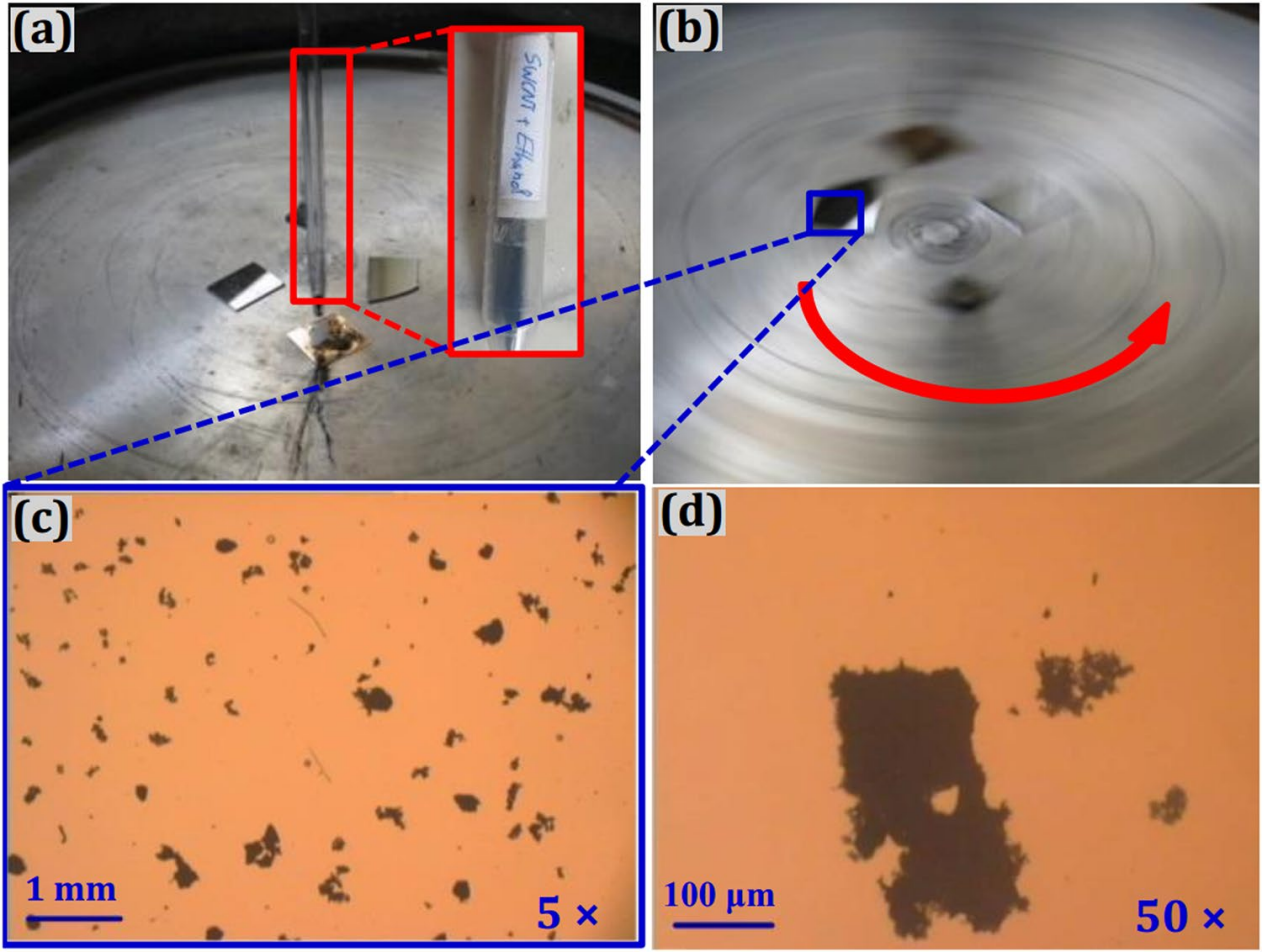
Spin coating process and the optical microscope images of nanotube clusters distributed on silicon substrates: (**a**) static dispense process, (**b**) spin-coating process, (**c**) microscope images 5×, and (**d**) microscope images 50×.

In this study, thin SWCNT films are prepared at the mechanical engineering center, University of Coimbra (Portugal) using spin-coating (or drop drying) method, and the spin-coating process is shown in Fig. 1. SWCNTs were prepared by arc discharge technique by the Int’tech Center, Kyoto University in Japan. The average diameter of the SWCNTs is around 1.2 nm. In the process of SWCNT thin films preparation, a dilute suspension of SWCNTs in ethanol was ultrasonicated for 20 min to spread out the nanotubes. The diffused suspension deposited on the well-polished silicon substrates of 1β0 mm × 10 mm using spin-coating method. At the beginning of the spin-coating process, a droplet of SWCNT suspension dropped on the substrates, a low spin speed of 200 rpm is used to spread the solution over the substrate, then a relatively high spin speed of 1000 rpm is accelerated to thin the samples to the final desired thickness within the appropriate rotational time, as shown in Fig. 1(b). Ethanol solvent was evaporated at room temperature. The samples were then subjected to heat treatment for 2 h under the temperature of 300 °C and slowly cooled down to room temperature. After heat treatment, nanotubes stuck and randomly oriented on the silicon substrate. The interactions of tube-to-tube and tube-to-substrate are through Van der Waals force. Figure 1(c) and (d) shows the optical microscope images of nanotube clusters distribution on the silicon substrates under 5 times magnification (c) and 50 times magnification (d).

The thickness of the deposited SWCNT film was obtained by a profile-meter technique. The surface profiling was carried out on the film edge. The detection of the scanned edge gives the projected wall thickness. The profile-meter indicates a SWCNTs film thickness of about 250 nm, the width of film is about 0.25 mm.

### Nanoidentation test

**Figure 2:**
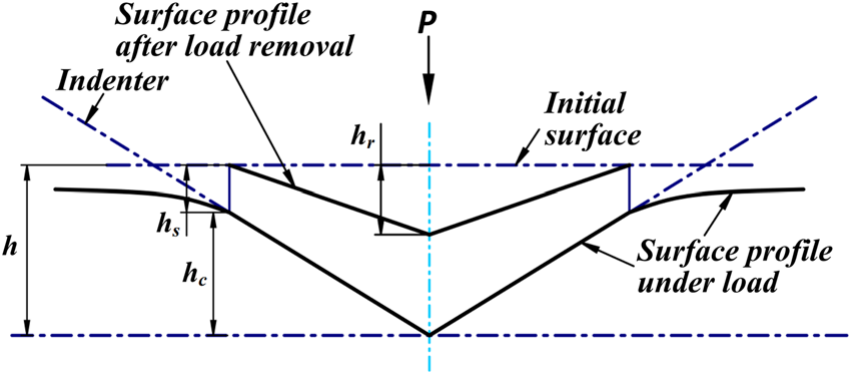
Typical indentation load-displacement curve.

Nanoindentation test measures the movement of a diamond probe in contact with the material surface. For indentation measurements, the indenter is impressed into the material surface under an increasing load; After it reaches a pre-determined maximum load or displacement, the load is reduced and the penetration depth decreasing due to the elastic recovery of the deformed material. Figure 2 shows the cross section of indentation. During indentation process, the displacements versus the applied loads are recorded through the precise actuator and sensor. Those records are then used to calculate the indentation hardness and elastic modules of the tested material. In nanoindentation technique, it is common to define the hardness of the material as the mean pressure under the load:

1 $$H=\frac{{P}_{{\rm{\max }}}}{A}$$

where *P*_max_ is the maximum load and *A* is the projected contact area at the maximum load which calculated from the contact depth *h*_*c*_^15^.

2 $$A=A({h}_{c})=24.5{h}_{c}^{2}+{C}_{1}{h}_{c}^{1}+{C}_{2}{h}_{c}^{1/2}+{C}_{3}{h}_{c}^{1/4}+\cdots +{C}_{8}{h}_{c}^{1/128}$$

**Figure 3:**
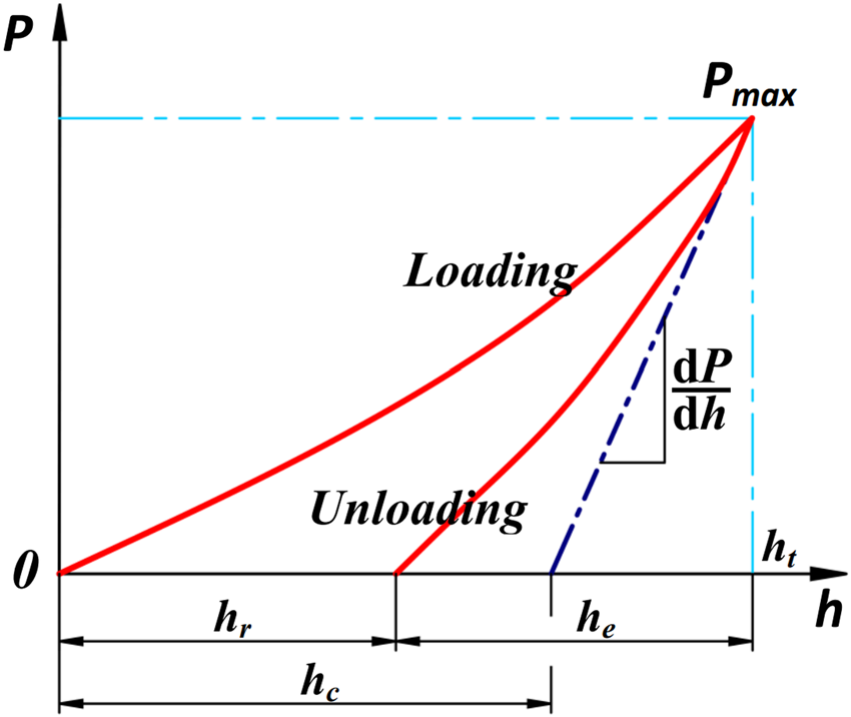
Nanoindentation system.

The Young’s modulus is obtained by contact mechanics:

3 $$\frac{1}{{E}_{r}}=\frac{(1-{v}^{2})}{E}+\frac{(1-{v}_{i}^{2})}{{E}_{i}}$$

where *E* and *v* are Young’s modulus and Poisson’s ratio of the tested material; *E*_*i*_ and *v*_*i*_ are the same parameters for the indenter; *E*_*r*_ is the reduced modulus which can be deduced from the initial slope of the unloading data (Fig. 3) as:

4 $${E}_{r}=\frac{1}{2}\frac{dp}{dh}\frac{\sqrt{\pi }}{\sqrt{A}}$$

**Figure 4:**
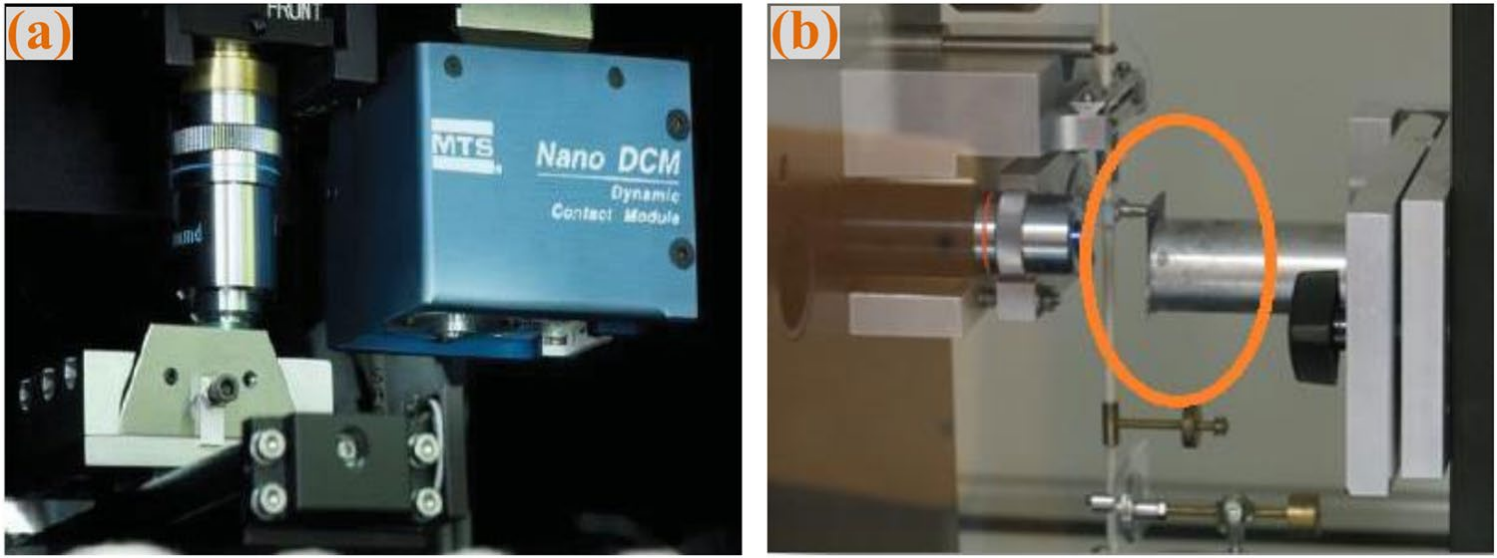
Reduced Modulus distribution.

Experimental tests for SWCNT film were performed at the nanoindentation platform system. The system has the load and displacement resolution of 1 nN and 0.0002 nm, respectively, the measurement range of film thickness is more than 200 nm. Berkovich indenter, a three side pyramid with a half angle of 65.3° was used in the experiment. The test surrounding temperature is controlled within 25 ± 1 °C, the humidity is about 63%. The test system was placed on a vibration free isolated chamber as shown in Fig. 4. The surface of the specimen is first scanned, and then a relatively large and uniform area of the film on the substrate was chosen for the test subject (Fig. 4(c)). The indenter was first loaded and unloaded three times successively at a constant rate to examine the reversibility of the deformation. Indentations were made at eighteen different nodes on the chosen area. Table 1 is the parameters setting in indentation procedure.

**Table 1:** Parameters setting in indentation procedure.

Parameters	Values
Maximum Load	3.00 mN
Limit stop load	0.15 mN
Initial load	0.05 mN
Loading rate	0.10 mN/s
Unloading rate	0.10 mN/s
Indentations	18
Dwell period at maximum load	5 s

## Experimental Results

Doerner observed that for some materials, the initial portions of unloading curves are linear^16^, and the unloading stiffness is then related the modulus and contact area through the relationship:

5 $$S=\frac{dP}{dh}=\frac{2}{\sqrt{\pi }}{E}_{r}\sqrt{A}$$

where, *S* = *dP*/*dh* is the initial unloading stiffness obtained by the initial portion of the unloading process. A is the projected area of the elastic contact. It is assumed that the contact area between the indenter and the material remains constant and moves elastically during unloading, and the plastic area is always equal to the contact area, and it is calculated using a polynomial function of order 2 in this study.

In this study, the experimental results are corrected for the thermal drift of equipment system. The hardness and elastic modulus are determined by using the method of power law fitting between 100% and 20% of the unloading data.

6 $$P=\alpha {(h-{h}_{f})}^{m}$$

where the constant *α*, *h*_*f*_, and *m* are determined by fitting the upper portion of unloading data. The indentation load-displacement data is analyzed according to equations (1) and (4). The elastic modulus of SWCNT film is then derived from equation (3) as:

7 $$E=(1-{v}^{2})/(\frac{1}{{E}_{r}}-\frac{1-{v}_{i}^{2}}{{E}_{i}})$$

**Figure 5:**
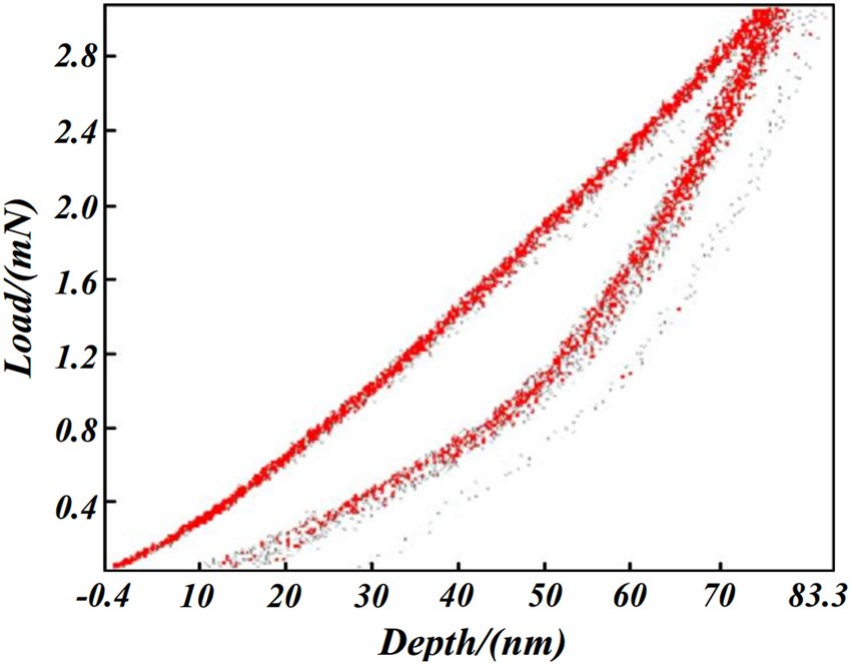
The overview of the experimental results.

**Figure 6:**
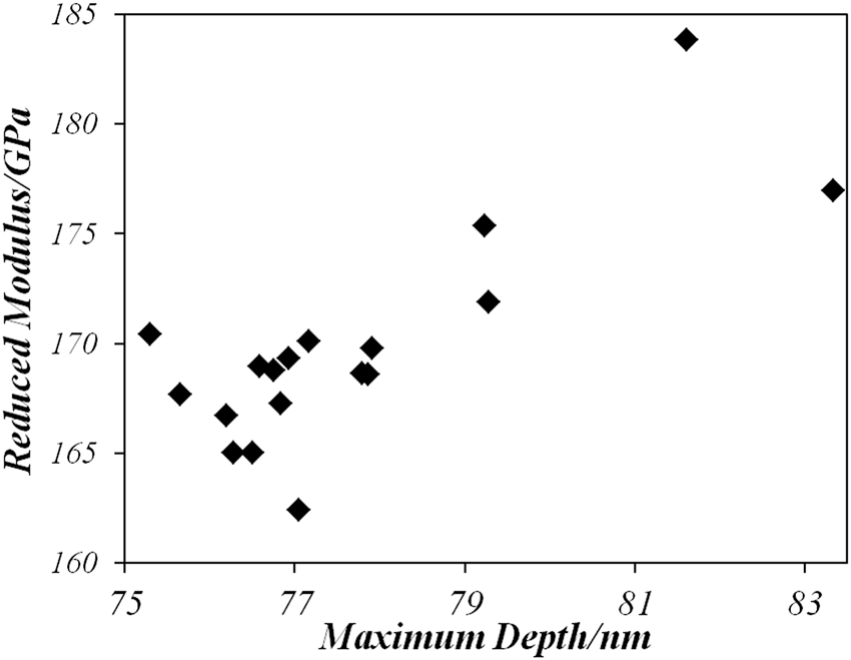
Reduced Modulus distribution.

Table 2 represents the averages test results of 18 indentations obtained automatically by the test system. The overview of the experimental results for the 18 groups of load-displacement curves is demonstrated in Fig. 5. And the Reduced modulus vs Maximum depth distribution which provided by the test system is illustrated in Fig. 6. The experimental loading-unloading curves show that there is one group of curve which is in bias with the bulk of the data. This inconsistent will be further discussed in the following uncertainty analysis part.

**Table 2:** Test results.

Parametres	Values
Maximum Load (nN)	3.054 ± 20.007
Maximum Depth (nm)	77.68 ± 2.06
Hardness (GPa)	12.577 ± 0.759
Reduced modulus (GPa)	169.818 ± 4.911

## Discussion

### Analysis of unloading curves

**Figure 7:**
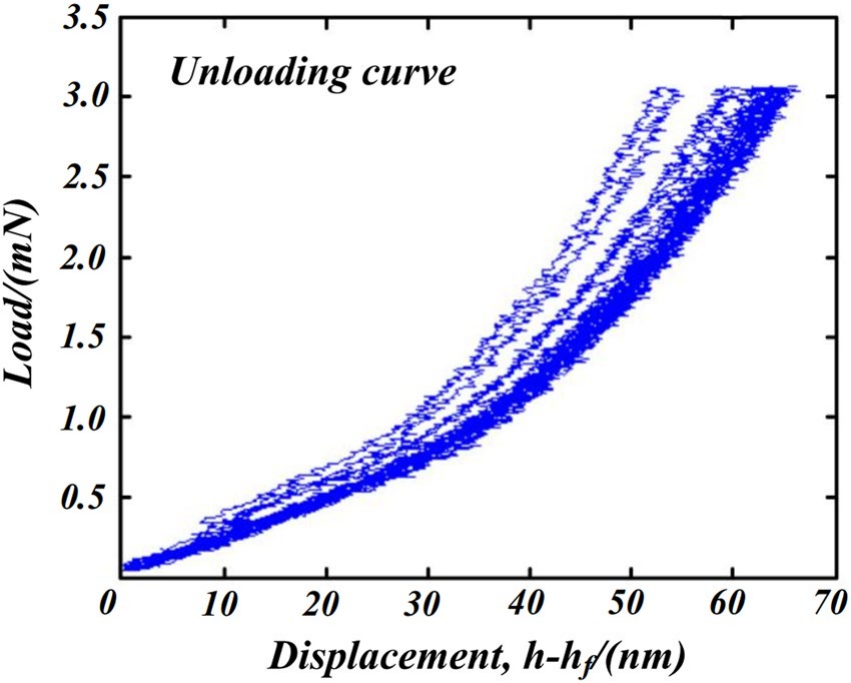
Unloading segment of the load-displacement data.

Errors estimation is a basic issue in the experiment uncertainty evaluation. In this study, the interest quantities of SWCNT film hardness *H* and reduced elastic modulus *E*_*r*_ are derived from the direct measurand of indentation loads and displacements. Parameters in the power law model (6) are obtained by making a power function regression analysis for the test data between 100% and 30% of unloading process, as shown in Fig. 7. The estimated parameters of model (6) for each set of indentation are given in Table 3.

**Table 3:** Estimated parameters by Power-law fitting.

Parameter	α	h_f_/(nm)	m	F_max_/(mN)	h_max_/(nm)
1	0.00133	16.97903	1.890	3.050	76.90
2	0.00130	18.58379	1.892	3.046	79.29
3	0.00144	14.20107	1.856	3.055	76.20
4	0.00230	22.86779	1.790	3.069	79.23
5	0.00152	27.89517	1.908	3.058	81.61
6	0.00213	18.23268	1.786	3.055	76.75
7	0.00193	17.27196	1.803	3.053	76.58
8	0.00189	19.64588	1.816	3.047	77.91
9	0.00140	16.22530	1.870	3.047	77.79
10	0.00148	28.73417	1.909	3.045	83.34
11	0.00184	16.06429	1.807	3.059	76.83
12	0.00188	19.11446	1.817	3.060	77.16
13	0.00208	18.56658	1.793	3.046	77.87
14	0.00149	14.38536	1.851	3.054	75.66
15	0.00123	12.92586	1.884	3.062	76.28
16	0.00146	14.64508	1.861	3.063	75.29
17	0.00129	14.38413	1.879	3.051	76.51
18	0.00169	16.12715	1.821	3.048	77.04
Mean	0.00165	18.15832	1.846	3.0538	77.68
Median	0.00151	17.1255	1.854	3.0535	76.97
Range	0.00107	15.8083	0.1229	0.024	8.05
Skewness	0.5147	1.293	0.004	0.51	1.5
Standard deviation	0.000322	4.4099	0.042	0.0069	2.058

### Parameters distribution analysis

**Figure 8:**
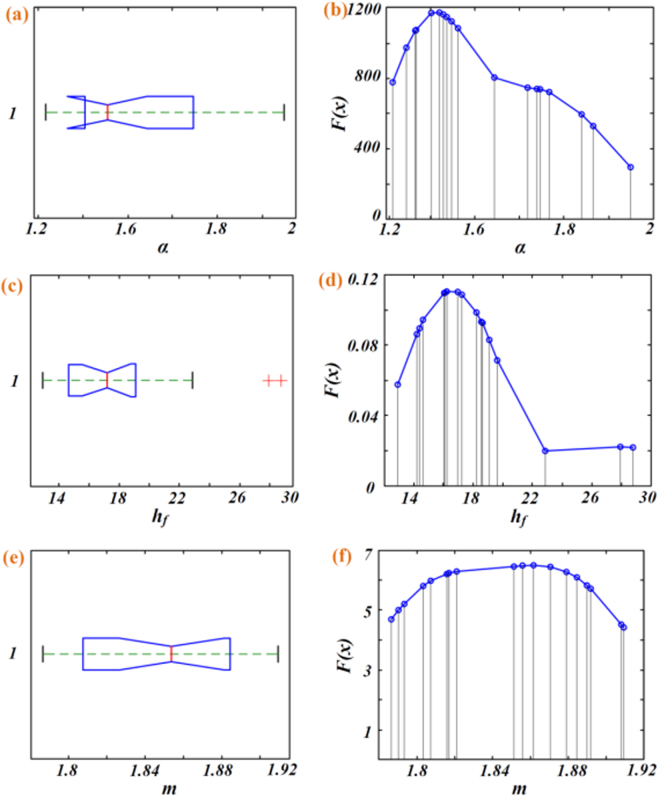
(**a,c,e)** boxplot of *α*, *h*_*f*_, and *m* in Table 3, (**b,d,f**); Pdf of *α*, *h*_*f*_, and *m*.

Data distribution of parameter *α*, *h*_*f*_, and *m* in Table 3 are characterized by distribution graph in Fig. 8. After analyzing the probability distribution of *α, h*_*f*_, and *m* we preliminarily hypothesize that *h*_*f*_ may obeys the lognormal distribution and *m* may obey the normal distribution, that is:

8 $$\mathrm{ln}\,\alpha  \sim N({\mu }_{\alpha },{\sigma }_{\alpha }^{2})$$

9 $$\mathrm{ln}\,{h}_{f} \sim N({\mu }_{{h}_{f}},{\sigma }_{{h}_{f}}^{2})$$

10 $$m \sim N({\mu }_{m},{\sigma }_{m}^{2})$$

By fitting the probability distribution of the values of *α*, *h*_*f*_, and *m* in Table 3 to the hypothesized distribution, *μ*_*α*_ and *σ*_*α*_ are equal to −6.42 and 0.19, respectively; and ${\mu }_{{h}_{f}}$ and ${\sigma }_{{h}_{f}}$ are equal to 2.87 and 0.22, respectively. Table 4 gives the 0.95 level of confidence interval for *h*_*f*_, and *m*.

**Table 4:** 95% confidence interval by parametric bootstrap method.

Parameter	α	h_f_	m
Mean	0.00165	18.15689	1.846
Confidence interval	[0.00112, 0.00214]	[10.71969, 25.28685]	[1.762, 1.930]

**Figure 9:**
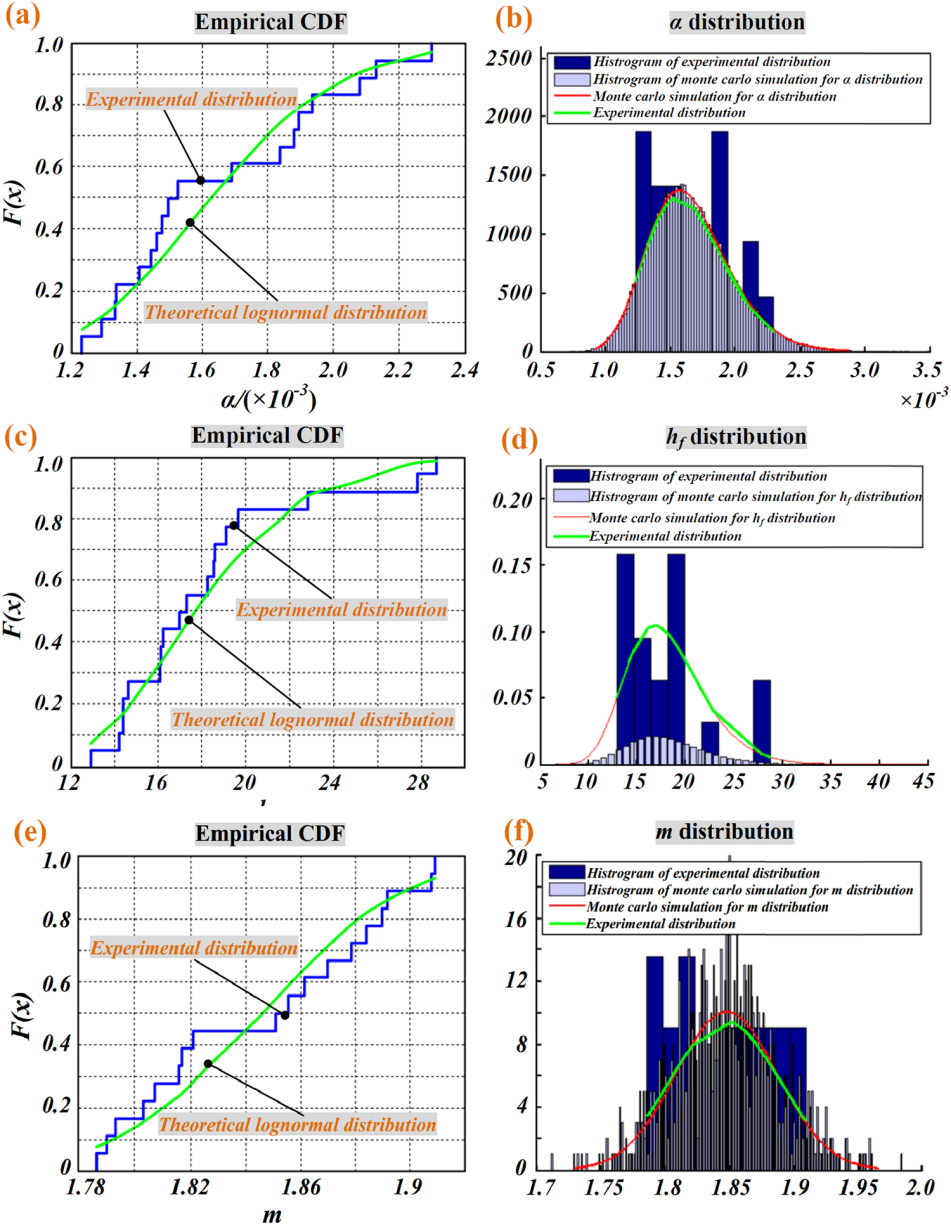
Parameter distribution. (**a**,**c**,**e**) The empirical CDF distribution and theoretical CDF distribution of the hypothesized function; (**b,d,f**) Comparison of empirical and theoretical fitted distribution.

For testing whether parameter *α, h*_*f*_ and *m* in Table 3 follow the expected probability distribution, a goodness of fit test by using the Kolmogorov-Smirnov and Monte Carlo simulation method are carried out. The comparison of empirical and theoretical fitted distributions for parameters distribution is illustrated in Fig. 9, it shows that the probability distributions of *α*, *h*_*f*_, and *m* fit the hypothesized distribution very well.

### Uncertainty analysis for unloading process curve

**Figure 10:**
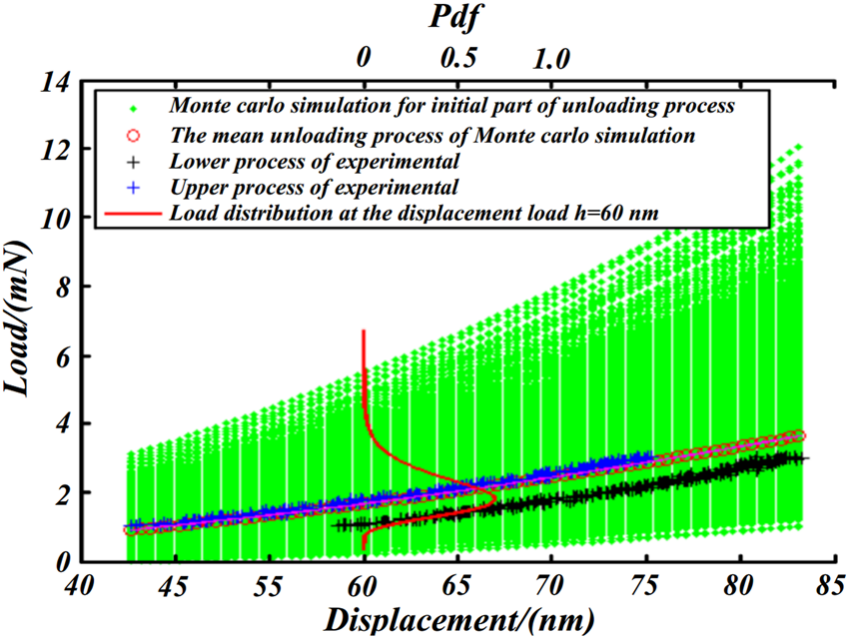
Comparison of the simulation and experimental load-displacement curve for unloading process.

Based on the statistic estimation of parameter *α*, *h*_*f*_ and *m* distribution we characterize the load-displacement curve for the 70% upper part of unloading process using Monte Carlo simulation method. Figure 10 is the comparison of the experimental and the simulation with a sample size of 5000 load-displacement curves for the upper part of unloading process. Comparison in Fig. 10 illustrates that experimental curves are rigorously inside the 95% confidence interval of the numerical simulation results, which demonstrates that the power law fitting model according well with the test results.

### Uncertainty estimation for Elastic modulus and hardness of SWCNT thin film

In equations (1) and (4), the contact area A is calculated from the contact depth *h*_*c*_, which is related to the total displacement *h*_max_ of indenter:

11 $${h}_{c}={h}_{{\rm{\max }}}-\varepsilon (\frac{{P}_{{\rm{\max }}}}{S})$$

here the contact stiffness *S* is equals to the tangent at the maximum load.

12 $${\rm{S}}=\frac{d{P}_{{\rm{\max }}}}{d{h}_{{\rm{\max }}}}=m\alpha {({h}_{{\rm{\max }}}-{h}_{f})}^{m-1}$$

The value of *ε* depends on the indenter geometry. For a Berkovich indenter *ε* is 0.75^17^.

### Area calibration and uncertainty evaluation

The area calibration relates the actual, non-ideal diamond contact area to the depth of penetration. The target of the area calibration is to find the function which can be used across a wide indentation range to obtain accurate hardness and modulus values. In this study, the diamond area *A* is calculated using a polynomial function of order 2:

13 $$A={c}_{2}{{h}_{c}}^{2}+{c}_{1}{h}_{c}+{c}_{0}$$

For an ideal Berkovitch indenter, *c*_2_ = 24.5, *c*_1_ = *c*_0_ = 0. A real Berkovitch indenter, although each diamond is slightly different, the typical values are *c*_2_ = 20~24, *c*_1_ = 1500~3000^17^. Here *c*_2_ = 21.93, *c*_1_ = 2330.6, and *c*_0_ = 52726.9.

According to the uncertainty propagation law, the standard area uncertainty *μ*(*A*) can be expressed as:

14 $${[\mu (A)]}^{2}=(2{c}_{2}{h}_{c}+{c}_{1})\times {[\mu ({h}_{c})]}^{2}+{\mu }_{{\rm{\Delta }}}^{2}$$

where ${\mu }_{{\rm{\Delta }}}^{2}$ is the residuals of fitting curve, and [*μ*(*h*_*c*_)] is the uncertainty of contact depth which can be derived from equation (11):

15 $${[\mu ({h}_{c})]}^{2}={[\mu ({h}_{max})]}^{2}+{[\frac{\varepsilon }{S}\mu ({F}_{max})]}^{2}+{[\frac{\varepsilon }{{S}^{2}}{F}_{max}\mu (S)]}^{2}$$

where *μ*(*h*_max_) is the uncertainty of maximum contact depth *μ*(*F*_max_) is the uncertainty of maximum applied load and *μ*(*S*) is the uncertainty of contact stiffness

16 $$\begin{array}{rcl}{[\mu (S)]}^{2} & = & m\alpha (m-1){({h}_{max}-{h}_{f})}^{m-2}[\mu {({h}_{max})}^{2}+\mu {({h}_{f})}^{2}]\,\\  &  & +m{({h}_{max}-{h}_{f})}^{m-1}[\mu (\alpha ){]}^{2}\,+\alpha ln(m-1)({h}_{max}-{h}_{f})[\mu (m){]}^{2}\end{array}$$

In this study, the standard maximum load uncertainty *μ*(*F*_max_) and the standard maximum contact depth uncertainty *μ*(*h*_mαx_) are calculated by the standard deviation of 18 nodes indentation test. As the parameter distribution analysis in section 4.1.1 stated that *α* and *h*_*f*_ follow a lognormal distribution the uncertainty are calculated as the root of the variance *u*(*α*) = 0.00032, *μ*(*h*_*f*_) = 4.024 nm, and *μ*(*m*) = 0.042, the uncertainty of *F*_max_ and *h*_max_ are *μ*(*F*_max_) = 0.0069 mN, *μ*(*h*_max_) = 0.4851 nm. So

17 $$\mu (S)=4.75\,\mathrm{nm}$$

### Uncertainty of hardness evaluation

The uncertainty of indentation hardness *μ*(*H*) can be obtained from equation (1):

18 $${[\mu (H)]}^{2}={[\frac{1}{A}\mu ({F}_{max})]}^{2}+{[\frac{{F}_{max}}{{A}^{2}}\mu (A)]}^{2}$$

Then the relative standard uncertainty of indentation hardness is:

19 $${[\frac{\mu (H)}{H}]}^{2}={[\frac{\mu ({F}_{max})}{{F}_{max}}]}^{2}+{[\frac{\mu (A)}{A}]}^{2}$$

### Uncertainty of reduced modulus evaluation

The uncertainty of reduced modulus *μ*(*E*_*r*_) can be obtained from equation (4)

20 $${[\mu ({E}_{r})]}^{2}={[\frac{\sqrt{\pi }}{2}\frac{1}{\sqrt{A}}\mu (S)]}^{2}+{[\frac{\sqrt{\pi }}{4}\frac{S}{{A}^{3/2}}\mu (A)]}^{2}$$

The relative standard uncertainty of reduced modulus can be calculated by

21 $${[\frac{\mu ({E}_{r})}{{E}_{r}}]}^{2}={[\frac{\mu (S)}{S}]}^{2}+{[\frac{1}{2}\frac{\mu (A)}{A}]}^{2}$$

The uncertainty analysis results reveal that the relative expanded uncertainty of hardness and reduced modulus corresponding to a level of confidence of 95% are separately 12.07% and 10.64%. The dispersion of hardness and reduced modulus are much larger than the tested values that automatically obtained by the indentation system in Table 2.

### Uncertainty of Young’s modulus evaluation

The Young’s modulus *E* is obtained from equation (7), which depends not only on the reduced modulus *E*_*r*_, but also on the modulus of indenter and the Poisson’s ratio of the sample. According to the literatures, Young’s modulus of Polycrystalline diamond changes from 1106 GPa to 1164 GPa, which depends on the orientation of structures. For the randomly orientated aggregates of diamond crystallites, it has the mean modulus and Poisson’s ratio of ${\bar{E}}_{i}$ = 1143 GPa, $\bar{v}$ = 0.07^18^. While, the Poisson’s ratio of SWCNT sheets depend on the inter-tube torsional angle and the tubes orientation to sheet plan^11^. The uncertainty of the Young’s modulus of SWCNNTs thin film *μ*(*E*) can be obtained from equation (7).

22 $$\begin{array}{rcl}{[\mu (E)]}^{2} & = & {[2v/(\frac{1}{{E}_{r}}-\frac{1-{v}_{i}^{2}}{{E}_{i}})\cdot \mu (v)]}^{2}\\  &  & +{[(1-{v}^{2})/{(\frac{1}{{E}_{r}}-\frac{1-{v}_{i}^{2}}{{E}_{i}})}^{2}\frac{1}{{{E}_{r}}^{2}}\cdot \mu ({E}_{r})]}^{2}\\  &  & +{[(1-{v}^{2})(1-{v}_{i}^{2})/{(\frac{1}{{E}_{r}}-\frac{1-{v}_{i}^{2}}{{E}_{i}})}^{2}\frac{1}{{{E}_{i}}^{2}}\cdot \mu ({E}_{i})]}^{2}\end{array}$$

The relative standard uncertainty of the Young’s modulus can be calculated by

23 $$\begin{array}{c}{[\frac{\mu (E)}{E}]}^{2}={[\frac{2v}{(1-{v}^{2})}\cdot \mu (v)]}^{2}+{[1/(\frac{1}{{E}_{r}}-\frac{1-{v}_{i}^{2}}{{E}_{i}})\frac{1}{{{E}_{r}}^{2}}\cdot \mu ({E}_{r})]}^{2}\\ +{[(1-{v}_{i}^{2})/(\frac{1}{{E}_{r}}-\frac{1-{v}_{i}^{2}}{{E}_{i}})\frac{1}{{{E}_{i}}^{2}}\cdot \mu ({E}_{i})]}^{2}\end{array}$$

Here, for a standard diamond indenter probe, $\bar{E}$_*i*_ = 1143 GPa, and $\bar{v}$_*i*_ = 0.07. Some researchers also take $\bar{E}$_*i*_ = 1100 GPa in literatures^14^. We suppose that the uncertainty of indenter’s Young’s modulus is about 0.038 percent at most, and the uncertainty is assumed to be uniformly distributed in the absence of better information, then the standard uncertainty of Young’s modulus *μ*(*E*_*i*_) = 24.83 GPa. According to Lee’s research^11^, we adopt *v* = 0.18, the uncertainty is assumed to be uniformly distributed, and the standard uncertainty of Poisson’s ratio *μ*(*v*) = 0.069.

The relative expanded standard uncertainty of the diamond area, hardness, reduced modulus, and Young’s modulus corresponding to a level of confidence of 95% are summarized in Table 5.

**Table 5:** The relative expanded standard uncertainty of parameters with a level of confidence of 95%.

Parameter	Relative expanded standard uncertainty
μ(A)	7.94%
μ(H)	12.07%
μ(E_r_)	10.64%
μ(E)	13.54%

## Conclusions

In this study, the elastic properties of SWCNT thin film (**~**250 nm) are studied by nanoindentation test. SWCNT thin films are prepared by the easy operated method of spin-coating technique and heat treatment. Hardness and young’s modulus of the SWCNT thin film are well tested. The uncertainty of hardness and Young’s modulus of SWCNT film is evaluated. The experimental results and uncertainty analysis reveal that the relative expanded uncertainty of hardness and Young’s modulus of SWCNT thin film corresponding to a level of confidence of 95% are separately 12.07% and 13.54%. The tested hardness and elastic modulus of SWCNT thin films are 12.577 ± 1.517 GPa and 192.83 ± 26.11 GPa, respectively. The uncertainty analysis indicates that nanoindentation test could be an effective and reliable technology in determining the mechanical properties of SWCNT thin film. This also suggests that nanoindentation technique could be a recommended method in determining properties of other nano scaled films.

## References

[1] Zhang D (2006). Transparent, conductive, and flexible carbon nanotube films and their application in organic light-emitting diodes. Nano Lett..

[2] Zhang M (2005). Strong, transparent, multifunctional, carbon nanotube sheets. Science.

[3] Teo KBK (2001). Uniform patterned growth of carbon nanotubes without surface carbon. Appl. Phys. Lett..

[4] Banerjee S, Hemraj-Benny T, Wong SS (2005). Covalent surface chemistry of single-walled carbon nanotubes. Adv. Mater..

[5] Wang, Q. & Moriyama, H. Carbon nanotube-based thin films: synthesis and properties, *Carbon Nanotubes-Synthesis, Characterization, Applications*. 487–514 (2011).

[6] Shima H (2012). Buckling of carbon nanotubes: a state of the art review. Materials.

[7] Tombler T (2000). Reversible electromechanical characteristics of carbon nanotubes under local-probe manipulation. Nature.

[8] Hall LJ (2008). Sign change of Poisson’s ratio for carbon nanotube sheets. Science.

[9] Ma YJ (2010). Carbon nanotube films change Poisson’s ratios from negative to positive. Appl. Phys. Lett..

[10] Schuh CA (2006). Nanoindentation studies of materials. Materialsloday.

[11] Li X, Bhushan B (2002). A. review of nanoindentation continuous stiffness measurement technique and its applications. Mater. Charact..

[12] Teodorescu M (2013). Experimental and theoretical investigations in polyamide spin-coated thin films. Mater. Plast..

[13] Qi HJ (2003). Determination of mechanical properties of carbon nanotubes and vertically aligned carbon nanotube forests using nanoindentation. J. Mech. Phys. Solids.

[14] Liu L, Cao G, Chen X (2008). Mechanisms of nanoindentation on multiwalled carbon nanotube and nanotube cluster. J. Nanomater..

[15] Oliver WC, Pharr GM (1992). An improved technique for determining hardness and elastic modulus using load and displacement sensing indentation experiments. Mater. Res. Soc..

[16] Doerner M, Nix W (1986). A method for interpreting the data from depth-sensing indentation instruments. J. Mater. Res..

[17] Beake BD (2002). Investigating the fracture resistance and adhesion of DLC films with micro-impact testing. Diam. Relat. Mater..

[18] Klein CA, Cardinale GF (1993). Young’s modulus and Poisson’s ratio of CVD diamond. Diam. Relat. Mater..

